# Wearable Localized Surface Plasmon Resonance-Based Biosensor with Highly Sensitive and Direct Detection of Cortisol in Human Sweat

**DOI:** 10.3390/bios13020184

**Published:** 2023-01-24

**Authors:** Minghui Nan, Bobby Aditya Darmawan, Gwangjun Go, Shirong Zheng, Junhyeok Lee, Seokjae Kim, Taeksu Lee, Eunpyo Choi, Jong-Oh Park, Doyeon Bang

**Affiliations:** 1 Korea Institute of Medical Microrobotics, 43-26 Cheomdangwagi-ro, Buk-gu, Gwangju 61011, Republic of Korea; 2 Robot Research Initiative, Chonnam National University, 77 Yongbong-ro, Buk-gu, Gwangju 61186, Republic of Korea; 3 School of Mechanical Engineering, Chonnam National University, 77 Yongbong-ro, Buk-gu, Gwangju 61186, Republic of Korea; 4 College of AI Convergence, Chonnam National University, 77 Yongbong-ro, Buk-gu, Gwangju 61186, Republic of Korea; 5 Graduate School of Data Science, Chonnam National University, 77 Yongbong-ro, Buk-gu, Gwangju 61186, Republic of Korea

**Keywords:** localized surface plasmon resonance, sweat biosensor, wearable sensor, aptamer, cortisol

## Abstract

Wearable biosensors have the potential for developing individualized health evaluation and detection systems owing to their ability to provide continuous real-time physiological data. Among various wearable biosensors, localized surface plasmon resonance (LSPR)-based wearable sensors can be versatile in various practical applications owing to their sensitive interactions with specific analytes. Understanding and analyzing endocrine responses to stress is particularly crucial for evaluating human performance, diagnosing stress-related diseases, and monitoring mental health, as stress takes a serious toll on physiological health and psychological well-being. Cortisol is an essential biomarker of stress because of the close relationship between cortisol concentration in the human body and stress level. In this study, a flexible LSPR biosensor was manufactured to detect cortisol levels in the human body by depositing gold nanoparticle (AuNP) layers on a 3-aminopropyltriethoxysilane (APTES)-functionalized poly (dimethylsiloxane) (PDMS) substrate. Subsequently, an aptamer was immobilized on the surface of the LSPR substrate, enabling highly sensitive and selective cortisol capture owing to its specific cortisol recognition. The biosensor exhibited excellent detection ability in cortisol solutions of various concentrations ranging from 0.1 to 1000 nM with a detection limit of 0.1 nM. The flexible LSPR biosensor also demonstrated good stability under various mechanical deformations. Furthermore, the cortisol levels of the flexible LSPR biosensor were also measured in the human epidermis before and after exercise as well as in the morning and afternoon. Our biosensors, which combine easily manufactured flexible sensors with sensitive cortisol-detecting molecules to measure human stress levels, could be versatile candidates for human-friendly products.

## 1. Introduction

The risk of several health conditions, such as high blood pressure, heart disease, diabetes, and mental illnesses is increased by stress, which is a sensation of emotional or physical tension triggered by self-awareness when the body encounters challenges or threats. Cortisol is a stress-related steroid hormone secreted by the adrenal cortex [1,2,3]. It also plays an important part in various physiological regulation processes, including those that control blood pressure, blood sugar, immune cells, inflammation, and carbohydrate metabolism in human blood, serum, saliva, and sweat [4,5,6]. Therefore, determining the cortisol levels in the body is quite important in both clinical settings and stress-related situations. Despite the widespread use of the current invasive methods of collecting blood samples to measure cortisol levels, the state of anxiety and fear in patients, as well as physical trauma and infection at the time of collection, can cause cortisol levels to change rapidly with emotion. Furthermore, lengthy detection processes and complicated detection equipment are also required to collect cortisol in body fluids, which eventually skews the results of the cortisol level analysis.

Consequently, it is envisaged that effective wearable sensors may be more suitable for monitoring cortisol levels in body fluids because wearable skin-interfaced biosensors provide a non-invasive way to detect the analytes quickly and accurately via direct skin contact, replacing traditional diagnostic methods that require complicated blood sampling [7,8]. Additionally, wearable biosensors’ ability to continuously monitor vital signs and provide feedback to the user makes them highly beneficial for the timely prevention, diagnosis, treatment, and management of diseases.

A broad range of analytical methods can be used to detect cortisol, such as the enzyme-linked immunosorbent assay (ELISA), Raman spectroscopy, chemiluminescence immunoassay, and chromatographic techniques [9,10,11,12]. However, the inability to detect cortisol levels is accompanied by several challenges, including limited sensitivity, long detection time, high cost, and procedural complexity. In contrast, the localized surface plasmon resonance (LSPR) technology is more beneficial for monitoring biomolecules than other techniques owing to its several advantages. Specifically, it offers a highly sensitive, selective, and label-free approach for the detection of biomolecules, as well as small sample size requirements, reusability, short experimental running time, stability under various ionic properties in sample solutions, low cost, electromagnetic (EM) interference resistance, and remote sensing capability [13,14,15]. Therefore, various approaches have been reported to develop plasmonic sensors with high sensitivities [16,17,18].

In this study, we developed a novel flexible LSPR biosensor. This flexible LSPR biosensor was manufactured by linking gold nanoparticles (AuNPs) to a 3-aminopropyltriethoxysilane (APTES)-functionalized poly (dimethylsiloxane) (PDMS) substrate for cortisol level detection in human sweat. The APTES was deposited on the O2 plasma-treated PDMS surface to fix the AuNPs to the PDMS substrate. PDMS is a widely used biocompatible substrate because it is cost-effective, non-toxic, flexible, and biocompatible. AuNPs can be immobilized on the surface of the APTES-modified PDMS through strong chemical interactions because amine groups have a high affinity to AuNPs [19]. The AuNPs were highly biocompatible and had unique LSPR characteristics, and their specific LSPR properties were affected along with their external environment variance. The cortisol aptamer was used to functionalize the surface of the prepared LSPR biosensor under optimized conditions owing to its high affinity to cortisol, small size, great stability, immobilization ability on the sensor surface, and high reproducibility. With the immobilization of the cortisol aptamer on the LSPR biosensor, this sensor can detect selective cortisol with high sensitivity [20]. This LSPR biosensor was shown to have the ability to detect cortisol concentrations in the range of 0.1–1000 nM, which correlates to sweat cortisol levels. The LSPR biosensor has a detection limit of 0.1 nM cortisol in sweat. Furthermore, the wearable LSPR sensor was directly attached to the human epidermis and was used to detect sweat cortisol levels before and after exercise in the morning and afternoon. After analyzing the LSPR biosensor samples by ultraviolet-visible spectroscopy (UV–Vis) for human sweat cortisol levels, the results of the sensor were correlated and validated with cortisol analysis using standard solutions. Our wearable LSPR biosensor could detect cortisol levels on the human epidermis directly and precisely.

## 2. Materials and Methods

### 2.1. Materials

PDMS SYLGARD 184 Silicone Elastomer base and a thermal curing agent were purchased from Dow Chemical Company (Midland, USA). Citrate-stabilized AuNP solution (80 nm in average diameter), 3-aminopropyltrimethoxysilane, tris-EDTA buffer solution (PH 7.4), and cortisol solution were purchased from Sigma Aldrich (Seoul, Republic of Korea). Cortisol aptamer containing a 5′-thiol modification was purchased from Bioneer (Daejeon, Republic of Korea) and purified by polyacrylamide gel electrophoresis (PAGE). The sequence of the cortisol aptamer was as follows:

5′-GGA ATG GAT CCA CAT CCA TGG ATG GGC AAT GCG GGG TGG AGA ATG GTT GCC GCA CTT CGG CTT CAC TGC AGA CTT GAC GAA GCT T-3′-thiol

All materials listed above were used without any additional purification.

### 2.2. Preparation of the Flexible Biosensor Based on PDMS-APTES AuNPs

**Figure 1:**
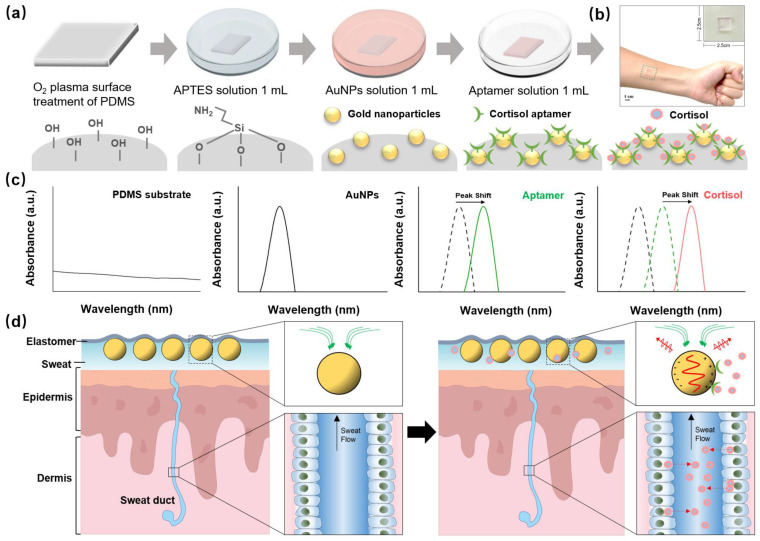
Schematic of the wearable sweat biosensor. (**a**) Fabrication process, (**b**) optical photography (**c**) reaction mechanism, and (**d**) structure of the human sweat gland showing the flexible LSPR biosensor interaction in the skin cross-section.

The PDMS substrate was prepared by mixing the PDMS monomer and its curing agent at a weight ratio of 10:1. Subsequently, this mixture was degassed under vacuum for 30 min at room temperature. After curing the PDMS for 2 h at 80 °C, the substrate was sliced into small pieces (10 mm × 10 mm). To immobilize AuNPs on the PDMS substrate, the pre-pared PDMS substrate was first treated with O2 plasma (10 min at 100 W) and then immersed in 1 mL of aqueous APTES (20%) solution for 30 min at room temperature. Thereafter, the substrate was rinsed with ethanol and distilled (DI) water to remove un-bound APTES. Subsequently, the amino-functionalized slide was immersed in 1 mL of AuNPs solution for 2 h and then washed with DI water. Finally, it was immersed in 1 mL of Tris-EDTA solution containing 100 nM of aptamer and washed with DI water (Figure 1a).

### 2.3. Characterization and Instrumentation

Scanning electron microscopy (SEM) and energy-dispersive X-ray (EDX) analysis (SU-8010, HITACHI Co., Tokyo, Japan) were used to analyze the surface topography of the AuNPs and AuNP-modified PDMS. High-resolution X-ray photo-electron spectroscopy (HR–XPS) (ESCA, VG Multilab 2000 system, UK), high-resolution X-ray diffraction (HR–XRD) (EMPYREAN, PANalytical Co., Almelo, The Netherlands), and Fourier-transform infrared spectroscopy (FT-IR) (Spectrum 400, PerkinElmer, Waltham, MA, USA) were used to determine the chemical compositions, crystallinity, and chemical interaction of the samples, respectively. For optical spectrum measurements, a sample with a size of 1 cm × 1 cm and a sensor area of 1 cm^2^ was placed in a plastic plate and the absorption spectrum was measured by a UV-Vis spectrometer (Thermo Fisher Scientific, Waltham, MA, USA). The scanning of the absorbance was between 400–800 nm. In the case of detecting human sweat, the prepared LSPR biosensor was stuck on the surface of skin for 10 min, and then measured by UV-Vis spectrometer. The statistical analyses were performed through Student’s *t*-test using the quantitative measurements of each sample group for at least four repetitions unless stated otherwise. The quantitative values were introduced as means and standard deviations. The symbol ** represents *p* < 0.01, and * represents *p* < 0.05, indicating that the result value was statistically significant.

## 3. Results

### 3.1. The Reaction Mechanism of Cortisol Detection in Human Sweat Using the Flexible LSPR Biosensor

LSPR is an optical phenomenon that occurs when light strikes metal nanoparticles. This is caused by the frequency matching between the incident light and the collective vibration of electrons on the surface of the metal nanoparticles [21,22]. The LSPR phenomenon exhibits high light absorption. Furthermore, the size, shape, and dielectric properties of each gold nanoparticle affect the LSPR as the LSPR changes in response to the external environment, thereby exhibiting various absorption properties. LSPR sensing begins with metal nanostructure surface binding of specific targets to induce plasmon peak shifts, which can be generally visualized by observing a red shift in the wavelength or a change in the extinction intensity of the peak with the binding of the target biomolecule.

The flexible LSPR biosensor consisted of AuNPs, which were fixed on the PDMS substrate (Figure 1c). PDMS is a versatile elastomer, and its chemical inertness, moldability, permeability, biocompatibility, and tunable mechanical nature make it an attractive candidate for our wearable LSPR biosensors. The surface of the PDMS is modified by APTES, allowing the AuNPs to be assembled on the PDMS surface using APTES as a linker.

The extinction peak of the LSPR spectra (green line) turned red when the cortisol aptamer was supplied to the flexible biosensor, indicating that it was less affected by other tiny molecules in sweat, allowing cortisol to be differentiated. A standard cortisol solution and real human sweat were used for the evaluation of the flexible LSPR biosensor, which revealed an extinction peak shift (red line) [23].

### 3.2. Morphological Study and Chemical Properties of the Flexible LSPR Biosensor

**Figure 2:**
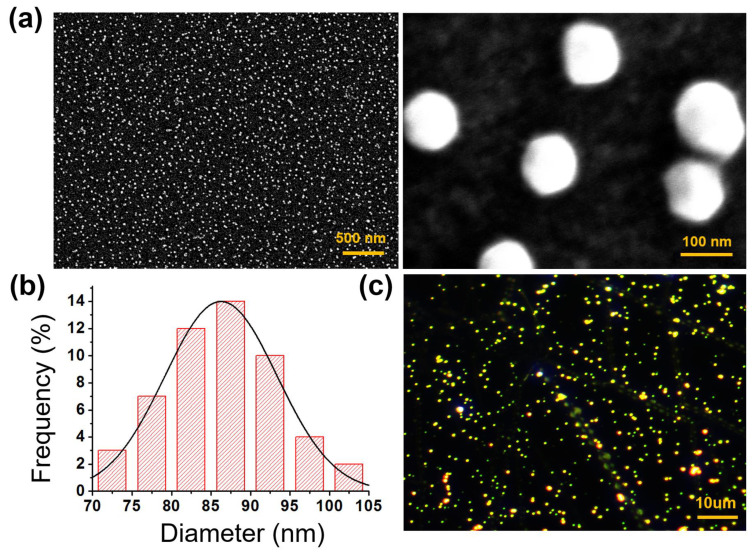
(**a**) SEM images, (**b**) frequency distribution curve, and (**c**) DFM of the flexible biosensor constructed using 80 nm AuNPs.

To fabricate the wearable LSPR biosensor, APTES was modified on the surface of PDMS after O_2_ plasma treatment, and the morphologies of AuNPs on PDMS were analyzed by SEM and dark-filed imaging [24,25,26]. As shown in Figure 2a, the monodispersed AuNPs were uniformly distributed (density~16.7 particles/µm^2^) on the PDMS substrate and were spherical without obvious aggregation, demonstrating that the APTES molecules were homogenously deposited on the PDMS substrate. This is essential for LSPR biosensing because AuNP aggregation weakens sensitivity. The size distribution curve of the AuNPs deposited on the PDMS substrate depicts an average diameter in the range of 86.268 ± 7.127 nm (Figure 2b). Figure 2c shows a dark-field image of the AuNPs placed on a PDMS substrate. The image was created by performing zero-order diffraction on a monochromator while the sample was illuminated with a broad light spectrum [27]. The SEM and dark-filed images show that the AuNPs were uniformly distributed on the PDMS substrate.

**Figure 3:**
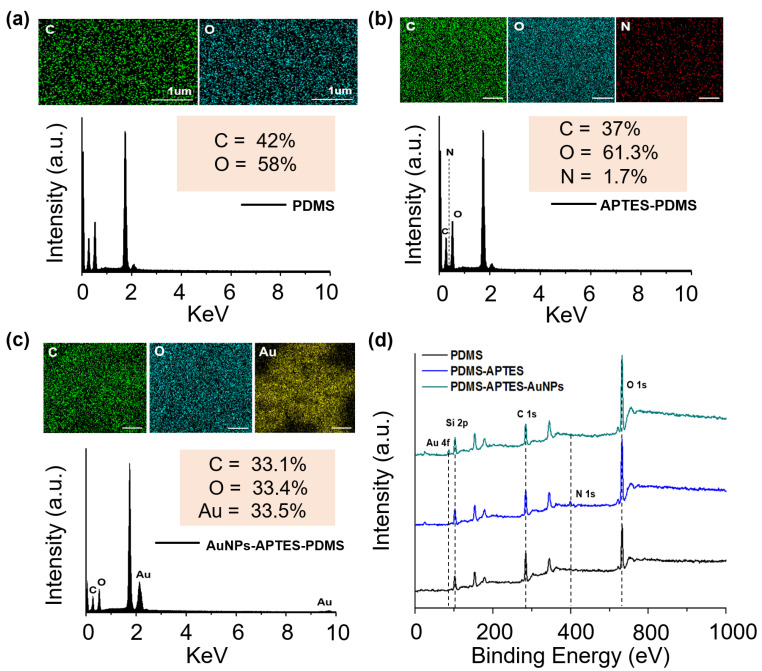
Characterization of the fabricated samples. EDX elemental analysis of (**a**) PDMS substrate, (**b**) PDMS-APTES, and (**c**) PDMS-APTES-AuNPs. (**d**) XPS curves of the LSPR biosensor.

To analyze the individual elements, EDX was performed on randomly selected portions on the surface of each sample to provide additional information regarding the chemical composition of the LSPR biosensor (Figure 3a–c). Peaks for the basic elements C and O were found on the PDMS substrate, while the N peak was newly presented in the APTES-PDMS substrate, indicating that the PDMS had been coated with APTES. Furthermore, the AuNPs were successfully coated on the APTES-PDMS substrate, as evidenced by the new Au peak and reduced carbon-to-oxygen ratio.

Using the peak at a specific wavenumber in the FT-IR, the existence of a specific functional group and chemical bond could be established (Figure S1 in Supplementary Materials). For the pristine PDMS spectrum, the symmetric and asymmetric stretching vibrations of C-H in the methyl groups were observed at 2902 and 2956 cm^−1^, respectively. The symmetric and asymmetric deformational vibrations of the -CH_3_ groups were attributed to the bands at 1260 cm^−1^ and 1410 cm^−1^ in the FT-IR spectra of the PDMS. Between 1000 cm^−1^ and 1100 cm^−1^, a large multicomponent peak resulting from symmetrical Si-O-Si stretching was also observed [28]. When compared to the spectrum of the pristine PDMS, a new characteristic peak at 3400–3600 cm^−1^ was observed. This was attributed to the O_2_ plasma treatment in the presence of the -OH band. After the APTES was coated on the PDMS substrate, a new N-H stretching at 3250–3400 cm^−1^ was observed, demonstrating the successful amine modification of the PDMS.

XPS analysis was used to further confirm the presence of AuNPs and APTES on the PDMS substrate. The XPS survey spectra of the substrate composited with PDMS, APTES-PDMS, and AuNPS-APTES-PDMS are shown in Figure 3d. In all cases, resolvable carbon (C 1s), oxygen (O 1s), and silicon (Si 2p) peaks were identified at 102, 284.5, and 532.5 eV, respectively. A new peak was observed at 400.5 eV (N 1s), indicating the existence of APTES on the PDMS substrate. After coating with AuNPs, a gold (Au 4f) peak was generated at 87.5 eV as a result of the successful anchoring of AuNPs on the APTES-modified PDMS substrate. These results show that the APTES was successfully immobilized on the surface of the stable PDMS substrate after the O_2_ plasma treatment. The AuNPs were also coated on the APTES-PDMS substrate owing to the strong interaction with APTES.

As illustrated in Figure S2, the XRD analysis of the AuNPs revealed the face-centered cubic (FCC) structure of gold and also confirmed that the AuNPs well-adhered to the APTES-functionalized PDMS substrate. The gold nanocrystals showed three unique peaks at 2*θ* = 38.08°, 44.62°, and 75.14°, which corresponded to conventional (111), (200), and (311) planes of the FCC lattice [29,30]. For the PDMS substrate, a sharp diffraction peak and an enormously broad diffraction peak appeared at 2*θ* = 12.08° and 23°, respectively [31]. There was no influence on the substrate after functionalizing the PDMS surface with APTES.

### 3.3. Characteristics of the LSPR Phenomenon

**Figure 4:**
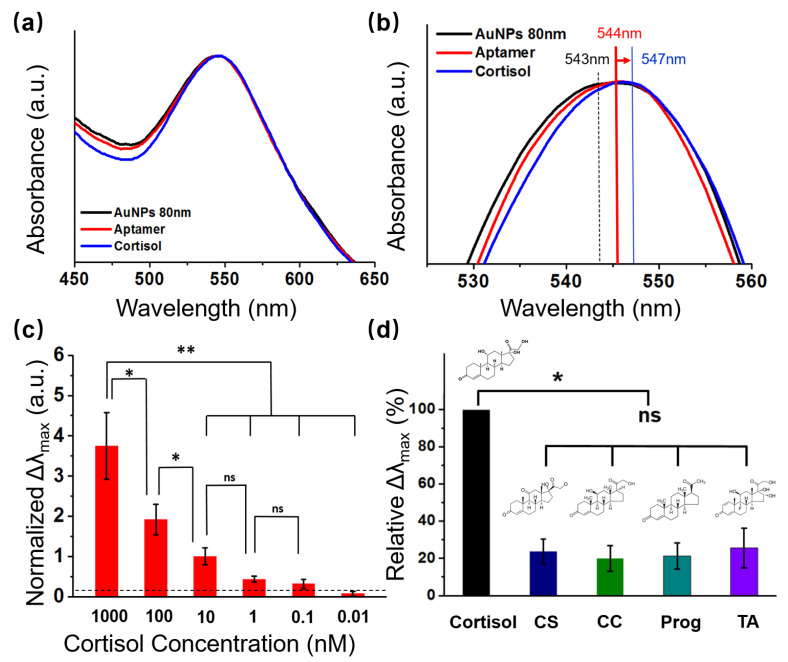
Absorption performance and selectivity test of the LSPR biosensors. (**a**) UV-Vis spectra showing shifting of the extinction peaks following aptamer and cortisol conjugation with 80 nm AuNPs; (**b**) magnified UV-Vis spectra showing the peaks shift, following aptamer and cortisol conjugation with AuNPs; (**c**) cortisol detection using the sweat biosensor at different concentrations of cortisol (0–1000 nM) (*n* = 4; * *p* < 0.05, ** *p* < 0.01, Student’s *t*-test); (**d**) selectivity test of the LSPR biosensor evaluated by comparing the relative maximum extinction peak shift (Δλmax) in response to 1 nM cortisol, cortisone (CS), corticosterone (CC), progesterone (Prog), and triamcinolone (TA) (*n* = 4; * *p* < 0.05, ** *p* < 0.01, ns = no significant difference, Student’s *t*-test).

When an analyte attaches to plasmonic nanostructures, the refractive index on the surface of the LSPR structure changes, causing the extinction peak to shift. The optical properties of the flexible sensors are determined by the size of the AuNPs. The UV-vis absorption spectrum of AuNPs with a diameter of 80 nm showed a distinct peak at about 545 nm (Figure 4a, black line), which corresponded to the LSPR peak of the AuNPs [32,33,34]. The LSPR peak of the aptamer-modified AuNPs was red-shifted from 543 nm to 544 nm, indicating that the biomolecules were immobilized on the AuNPs surface. The change in extinction peak after 1000 nM of cortisol was applied to the aptamer-modified AuNPs is depicted in Figure 4a,b (blue line). In this experiment, the peak of the cortisol-aptamer-AuNPs shifted from 544 nm to 547 nm, demonstrating that cortisol was effectively detected by the flexible LSPR biosensor.

### 3.4. Cortisol Analysis Using Standard Solutions

To examine the sensitivity of our wearable LSPR biosensor, various concentrations of cortisol solution (0.01, 0.1, 1, 10, 100, and 1000 nM) were monitored by the LSPR spectra variance of the presented biosensor (Figure 4c and Figure S3). The refractive index changes in the LSPR spectrum are illustrated by the shift in the absorption peak (Δλmax) of the LSPR spectrum after completion of the interactions with cortisol solution at concentrations of 0.01, 0.1, 1, 10, 100, and 1000 nM. Δλmax was normalized by dividing the absorption peak before and after the cortisol reaction by the value before the cortisol reaction.

The equation is shown as follows:

Normalized Δλ_max_ = (Δλ_max_(after cortisol reaction) − Δλ_max_(before cortisol reaction))/Δλ_max_(before cortisol reaction) (1)

The signals were gradually strengthened as the cortisol level increased from 0 nM to 1000 nM. Additionally, as the concentration of cortisol increased, the normalized Δλmax value of the LSPR flexible biosensors also increased. These results suggest that changes in the biosensor normalized Δλmax value at a specific wavelength can be used to determine the concentration of human sweat, according to the observed linearity (1–1000 nM).

### 3.5. Selectivity Test of Cortisol

To assess the specificity of the LSPR biosensor, we compared the relative variations in the maximum absorbance peak of several steroid hormones that share structural similarities with cortisol, such as cortisone (CS), corticosterone (CC), progesterone (Prog), and triamcinolone (TA), which were in response to 1 nM cortisol.

The corresponding values of the relative Δλmax were calculated based on the following equation:

Δλ_max_ (%) = Δλ_max(interfering molecules)/_Δλ_max(cortisol)_ × 100 (2)

where Δλ_max(interfering molecules)_ denotes the maximum wavelength shift reasoned by the addition of each type of interfering molecule and Δλ_max(cortisol)_ denotes the maximum wavelength shift reasoned by cortisol [35]. From the redshift in the LSPR spectra in Figure 4d, a significant difference can be observed in the LSPR spectra results obtained from the four other substances tested with a similar chemical structure to cortisol, suggesting that the cortisol can be specifically detected by the LSPR biosensor.

### 3.6. Cortisol Analysis Using Standard Solutions

**Figure 5:**
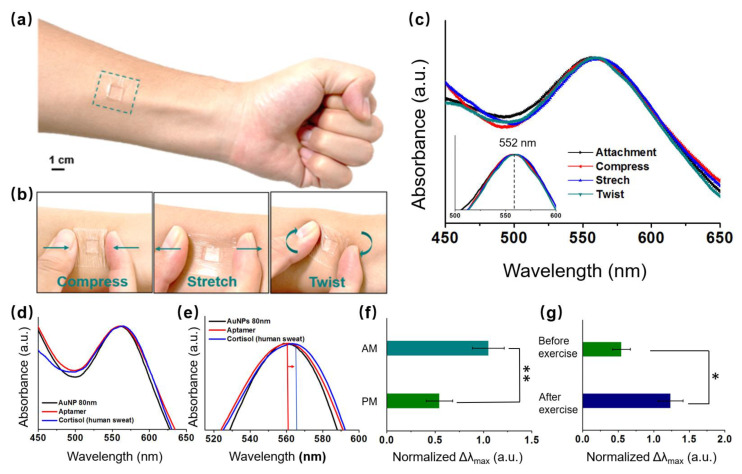
Schematic of the sweat biosensors (**a**) attached, (**b**) compressed, stretched, and twisted on the skin to extract cortisol. (**c**) UV-Vis spectra of the extinction peaks of the LSPR biosensor at the different statuses (Figure 4a,b). (**d**) UV-Vis spectra of the extinction peaks following aptamer and cortisol (from human skin) conjugation with 80 nm AuNPs. (**e**) UV-Vis spectra of magnified extinction spectra, following aptamer and cortisol conjugation with AuNPs. The cortisol levels of the LSPR flexible biosensor in the human epidermis (**f**) in the morning and afternoon, and (**g**) before and after exercise (n = 4; * *p* < 0.05, ** *p* < 0.01, Student’s *t*-test).

We validated that our wearable LSPR biosensor could detect endogenous circulating cortisol levels from human sweat samples (sweat samples collected from three healthy subjects). A schematic of a 1.0 cm × 1.0 cm flexible LSPR biosensor attached to the skin for sweat collection is shown in Figure 1b. Owing to their breathability and conformation to the skin, adhesive TegadermTM transparent dressings (2.5 cm × 2.5 cm) were employed to firmly adhere the biosensor to the skin site [7,36]. Prior to detection, the stability of the flexible LSPR sensor was investigated through various mechanical deformations of Tegaderm, such as attachment, compression, stretch, and twist, as illustrated in the schematic in Figure 5a,b. The corresponding UV-Vis spectra of the extinction peaks are shown in Figure 5c. There was no obvious difference of LSPR peak location when the biosensor detected the cortisol in sweat in the attachment, compress, stretch, and twist phases. Thus, the biosensor was unaffected by slight deformations and remained stable. The cortisol levels in the human sweat sample were subsequently analyzed after our flexible LSPR sensor was directly attached to the human skin (Figure 5d,e). Similar to the previous results with cortisol solutions, the extinction peaks were shifted by identifying the cortisol. Therefore, the LSPR biosensors achieve stable and reliable results even when people move while in use.

It is normally released in a 24 h cycle, peaking in the morning and gradually decreasing into the afternoon and evening [37,38]. The correlation between sweat and circulating cortisol levels was further understood by analyzing sweat samples from three healthy subjects in the early morning (AM) and late afternoon (PM) (Figure 5f). The cortisol levels were 2–3 times higher in the morning than in the afternoon, and this was consistent with the previously mentioned normal phenomenon. Additionally, variations in cortisol levels in the sweat samples during afternoon exercises compared to rest time were also investigated. The sweat cortisol levels during the day exhibited distinct baselines before stimulus (Figure 5g). Exercise causes the body to experience stress, which in turn triggers the release of cortisol. In reaction to the physiological stressor, significantly higher cortisol levels were observed in the sample without exercise compared to that with 50 min exercise [39,40]. Moderate to high-intensity exercise causes increases in cortisol levels, owing to a combination of hemoconcentration and HPA axis stimulus (ACTH), which can be effectively detected by our LSPR biosensor.

## 4. Discussion

In this study, we developed a flexible LSPR biosensor by depositing AuNP layers on an APTES-functionalized PDMS substrate. Owing to the advantages of cost-effective production, low immunogenicity, and high stability, cortisol-selective aptamers were used to modify the surface of the AuNP-modified PDMS substrate to create a highly selective and specific LSPR biosensor. Cortisol molecules interact with the surface of the sample as they come into contact with it, changing the way the aptamer molecules are recognized and, consequently, the LSPR spectra of the AuNPs on our wearable biosensor. The flexible LSPR biosensor exhibits excellent detection ability in cortisol solutions with varying concentrations ranging from 0.1–1000 nM, with a detection limit of 0.1 nM. The wearable LSPR biosensor also exhibits a high stable performance without being affected by slight deformation during the attachment, compression, and torsion phases. Additionally, the cortisol levels of the wearable LSPR biosensor were also measured in the human epidermis before and after exercise in the morning and afternoon. The cortisol concentration detection range was 100 nM in the morning and 1 nM in the afternoon, or 1 nM before exercise and 100 nM after exercise. This wearable LSPR biosensor also exhibits high stability under various mechanical deformations. With its remarkable performance, we believe that the developed flexible LSPR biosensor will pave the path for effectively advancing the human health monitoring field.

## Data Availability

The data presented in this study are available on request from the corresponding author.

## Supplementary Materials

The following supporting information can be downloaded at: https://www.mdpi.com/article/10.3390/bios13020184/s1, Figure S1: FT-IR spectra of PDMS, PMDS-O2 plasma, PDMS-(O2)-APTES, and PDMS-(O2)-APTES-AuNPS; Figure S2: XRD analysis of PDMS, PDMS-APTES, PDMS-APTES-AuNPs; Figure S3: Cortisol detection using the sweat biosensor at different concentrations of cortisol.
