# Supplementary material for: Wearable Localized Surface Plasmon Resonance-Based Biosensor with Highly Sensitive and Direct Detection of Cortisol in Human Sweat
Source: Biosensors (Basel). 2023 Jan 24;13(2):184. doi: 10.3390/bios13020184 (PMC9953546; doi:10.3390/bios13020184)
Supplement: Supplementary file 1 [file biosensors-13-00184-s001.zip › biosensors-2107872-supplementary.pdf]

biosensors

MDPI

Supplementary material

Wearable Localized Surface Plasmon Resonance-Based Biosensor with Highly Sensitive and Direct Detection of Cortisol in Human Sweat

Minghui Nan [1,2], Bobby Aditya Darmawan [1], Gwangjun Go [1], Shirong Zheng [1,3], Junhyeok Lee [1,3], Seokjae Kim [1,3], Taeksu Lee [1,*], Eunpyo Choi [1,2,3,4,*], Jong-Oh Park [1,*] and Doyeon Bang [1,2,4,5,*]

1 Korea Institute of Medical Microrobotics, 43-26 Cheomdangwagi-ro, Buk-gu, Gwangju 61011, Republic of Korea
2 Robot Research Initiative, Chonnam National University, 77 Yongbong-ro, Buk-gu, Gwangju 61186, Republic of Korea
3 School of Mechanical Engineering, Chonnam National University, 77 Yongbong-ro, Buk-gu, Gwangju 61186, Republic of Korea
4 College of AI Convergence, Chonnam National University, 77 Yongbong-ro, Buk-gu, Gwangju 61186, Republic of Korea
5 Graduate School of Data Science, Chonnam National University, 77 Yongbong-ro, Buk-gu, Gwangju 61186, Republic of Korea
* Correspondence: tslee@kimiro.re.kr (T.L.); eunpyochoi@jnu.ac.kr (E.C.); jop@kimiro.re.kr (J.-O.P.); db@jnu.ac.kr (D.B.)

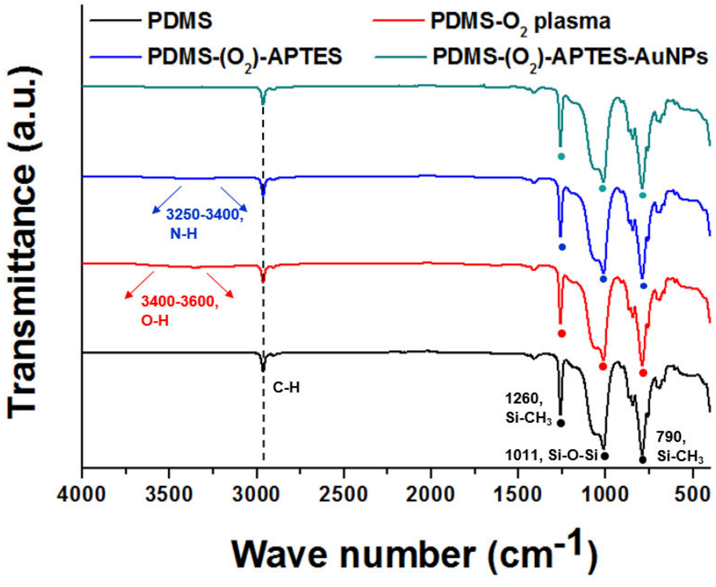

Figure S1. FT-IR spectra of PDMS, PMDS-O2 plasma, PDMS-(O2)-APTES, and PDMS-(O2)-APTES-AuNPS.

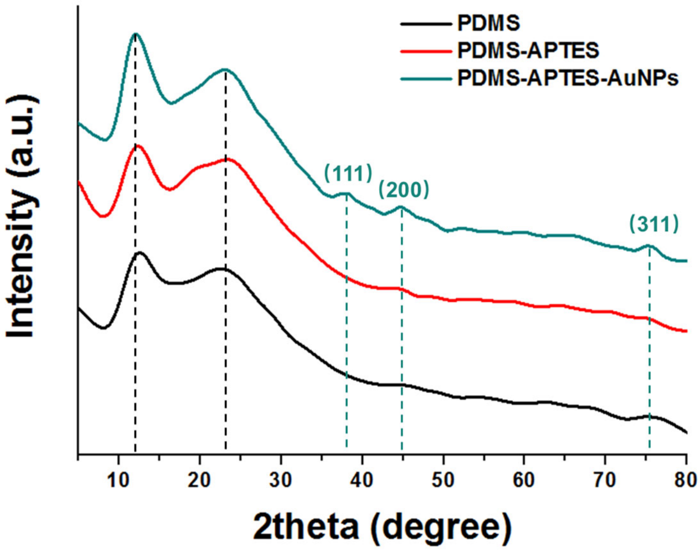

Figure S2. XRD analysis of PDMS, PDMS-APTES, PDMS-APTES-AuNPs.

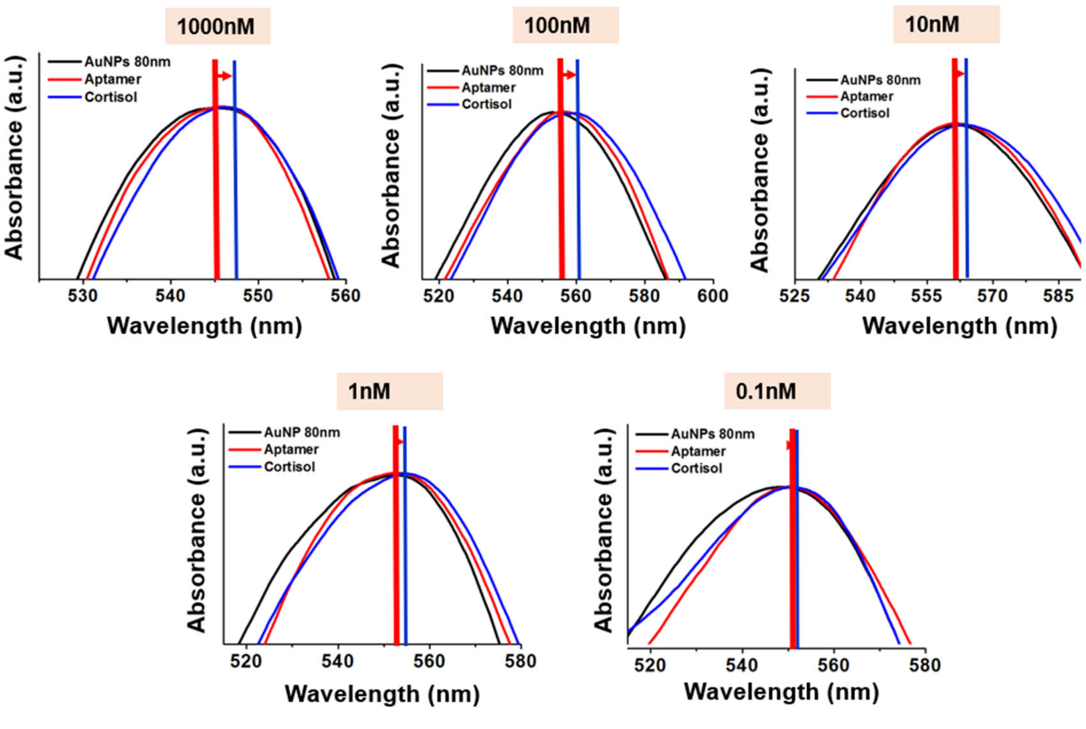

Figure S3. Cortisol detection using the sweat biosensor at different concentrations of cortisol.
